# Effect of Fluoride on the Morphology and Electrochemical Property of Co_3_O_4_ Nanostructures for Hydrazine Detection

**DOI:** 10.3390/ma11020207

**Published:** 2018-01-29

**Authors:** Tuantuan Zhou, Wanlin Gao, Qiang Wang, Ahmad Umar

**Affiliations:** 1 College of Environmental Science and Engineering, Beijing Forestry University, 35 Qinghua East Road, Haidian District, Beijing 100083, China; tuantuanzhou@bjfu.edu.cn or ztuant86@126.com (T.Z.); gaowanlin2016@bjfu.edu.cn (W.G.); 2 Department of Chemistry, College of Science and Arts, and Promising Centre for Sensors and Electronic Devices (PCSED), Najran University, Najran 11001, Saudi Arabia

**Keywords:** cobaltosic oxide, fluoride, precursor, crystallinity, electrochemical activity

## Abstract

In this paper, we systematically investigated the influence of fluoride on the morphology and electrochemical property of Co_3_O_4_ nanostructures for hydrazine detection. The results showed that with the introduction of NH_4_F during the synthesis process of Co_3_O_4_, both Co(CO_3_)_0.5_(OH)·0.11H_2_O and Co(OH)F precursors would be generated. To understand the influence of F on the morphology and electrochemical property of Co_3_O_4_, three Co_3_O_4_ nanostructures that were respectively obtained from bare Co(CO_3_)_0.5_(OH)·0.11H_2_O, Co(OH)F and Co(CO_3_)_0.5_(OH)·0.11H_2_O mixtures and bare Co(OH)F were successfully synthesized. The electrochemical tests revealed the sensing performance of prepared Co_3_O_4_ nanostructures decreased with the increase in the fluoride contents of precursors. The more that dosages of NH_4_F were used, the higher crystallinity and smaller specific surface area of Co_3_O_4_ was gained. Among these three Co_3_O_4_ nanostructures, the Co_3_O_4_ that was obtained from bare Co(CO_3_)_0.5_(OH)·0.11H_2_O-based hydrazine sensor displayed the best performances, which exhibited a great sensitivity (32.42 μA·mM^−1^), a low detection limit (9.7 μΜ), and a wide linear range (0.010–2.380 mM), together with good selectivity, great reproducibility and longtime stability. To the best of our knowledge, it was revealed for the first time that the sensing performance of prepared Co_3_O_4_ nanostructures decreased with the increase in fluoride contents of precursors.

## 1. Introduction

Hydrazine and based chemicals are water soluble volatile colorless liquids, and the simplest unique diamine in its class has aroused wide concern for its large number of applications in many spheres, for instance, corrosive inhibitors, fuel cells and so on [1,2,3,4]. The laboratory research and commercial application of hydrazine as a reducing agent and catalyst are commonly implemented. However, hydrazine and its derivatives do great harm to the body through the digestive system along with skin permeation [5]. Consequently, it is highly imperative to propose a sensitive, original and analytically credible tool for the effective detection of hydrazine. Recently, electroanalytical techniques have developed as a desirable method for detection of many chemicals, such as hydroquinone [6], acetone [7], herbicides [8], etc. due to their great sensitivity, better efficiency and low cost. It is promising to develop electrochemical methods for hydrazine detection. Nowadays, lots of semiconductor metal oxides have been used for hydrazine electrochemical sensing. Ahmad et al. have fabricated a ZnO nanorods-based hydrazine sensor and showed a low detection limit [9]. Wu et al. used MnO_2_ nanoflowers for hydrazine detection and exhibited a high sensitivity [10]. However, the study on Co_3_O_4_ utilized for hydrazine is rare to report.

Cobaltosic oxide (Co_3_O_4_), a kind of vital transition-metal oxide semiconductor with direct optical band gaps at 2.19 eV [11], has undergone extensive exploration recently for its wide-ranging potential applications as a gas sensor [12,13,14], catalyst [15,16,17,18], magnetic material [19], solar-energy absorber [20], supercapacitors and rechargeable lithium-ion-battery materials [21,22,23,24]. More significantly, because of its high catalytic performance, nanostructured Co_3_O_4_ is widely considered to be an attractive modified electrode material that can enhance the rate of electron transfer and minimize its over potential [25]. Up to now, various synthetic protocols have been developed for the synthesis of Co_3_O_4_ with diverse morphologies and designed nanostructures. Among them, a two-step approach, where co-based intermediates, such as Co(OH)_2_ and (Co(CO_3_)_0.5_(OH)·0.11H_2_O), are first fabricated by thermal annealing exposed to air, as the control on the morphologies of these intermediates has been widely used. However, for certain synthesis procedures, NH_4_F was normally used during the synthesis step, because F is a good complexing ligand for Co^2+^. In the presence of F^−^, CoF^+^ complex can firstly be formed to prevent Co(OH)_2_ generation, which would make the morphology controllable [26,27]. Meanwhile, F may also lead to the formation of (Co(OH)F), which may cause the generation of impurities in the products. Up to now, the influence of F content on the formation and electrochemical performance of Co_3_O_4_ has not been clarified.

Thus, in this paper, the influence of fluoride (F) on the morphological and electrochemical performance of Co_3_O_4_ for hydrazine sensing was systematically investigated for the first time. Three types of Co_3_O_4_ samples were synthesized by thermal treatment of their precursors, which contain different amounts of F. The physical and chemical compositions of all constructed materials were systematically investigated. The electrochemical activity of these Co_3_O_4_ were comprehensively compared using cyclic voltammetry and amperometric response analysis.

## 2. Results and Discussion

### 2.1. Structural and Morphological Studies

**Figure 1:**
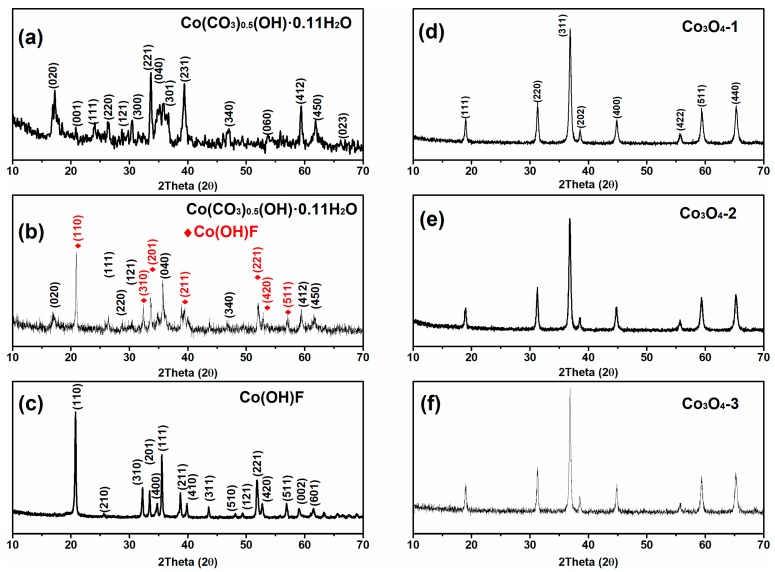
XRD patterns of (**a**) Co(CO_3_)_0.5_(OH)·0.11H_2_O; (**b**) Co(CO_3_)_0.5_(OH)·0.11H_2_O and Co(OH)F mixture; (**c**) Co(OH)F; (**d**) Co_3_O_4_-1; (**e**) Co_3_O_4_-2 and (**f**) Co_3_O_4_-3.

The composition of the prepared precursors was first examined by XRD characterization. As shown in Figure 1a,c, all of the diffraction peaks could be well indexed to Co(CO_3_)_0.5_(OH)·0.11H_2_O (JCPDS card No. 48-0083) and Co(OH)F (JCPDS card No. 50-0827), respectively, and no other peak could be found from the XRD patterns. Figure 1b verifies that the precursor powders are the mixture of Co(CO_3_)_0.5_(OH)·0.11H_2_O and Co(OH)F. Furthermore, the characteristic peaks at 17.5°, 24.2°, 26.8°, 28.8°, 35.5°, 59.9°, and 62.2° can be indexed to the reflections of (020), (111), (220), (121), (040), (340), (412), and (450) planes of Co(CO_3_)_0.5_(OH)·0.11H_2_O; while the characteristic peaks at 20.8°, 32.3°, 33.5°, 38.8°, 51.9°, 52.8°, and 57.0° can be indexed to the reflections of (110), (310), (201), (211), (221), (420), (511) planes of Co(OH)F. For the synthesis of Co_3_O_4_, all these three precursors were thermally annealed at 400 °C for 5 h in air. Figure 1d–f clearly indicate that all the diffraction peaks are well-assigned to the standard diffraction pattern of Co_3_O_4_ (JCPDS card No. 43-1003), which indicates that the precursors had been thoroughly converted to Co_3_O_4_ phase and there were no other impurities that could be detected. The crystallinity of the prepared products was evaluated via the Formula (1).

*X*c = *I*c/(*I*c + *KI*a) × 100% (1)

In Formula (1), *X*c represents the crystallinity measured by X-ray diffractometry; *I*c is the integral intensity of the crystal diffraction peak; and *I*a is the integral intensity of the amorphous diffuse peak; *K* is the elative scattering factor.

The crystallinity of prepared Co_3_O_4_ samples is calculated and shown in Table 1. It is obvious that Co_3_O_4_-1 possesses the lowest crystallinity (73.87%) and Co_3_O_4_-3 has the highest crystallinity 91.62%). To explore the influence of F on the crystallinity of Co_3_O_4_, different dosages of NH_4_F (from 0 to 20 mmol) had been added during the synthesis process of Co_3_O_4_. As shown in Table 1, the crystallinity of Co_3_O_4_ presents an increase trend with increasing the amount of NH_4_F during the synthesis process of precursors, which indicates that the strong interaction between Co^2+^ and F^−^ can make the particles grow regularly.

The formation of the precursors and annealed products can be illustrated with the following steps. When CO(NH_2_)_2_ is used as a hydrolysis reagent, the formation process of Co(CO_3_)_0.5_(OH)·0.11H_2_O can be depicted as Equations (2)–(4). While when HMT is used as a hydrolysis reagent, with the existence of F^-^, the formation of Co(OH)F can be possibly expressed by Equations (5)–(8). After calcination, both of the precursors can be converted into cobaltosic oxide by Equations (9) and (10).

H_2_O + CO(NH_2_)_2_ → CO_2_ + 2NH_3_ (2)

H_2_O + CO_2_ → 2H^+^ + CO_3_^2^^−^ (3)

0.11H_2_O + Co^2+^ + OH^−^ + 0.5CO_3_^2^^−^ → Co(CO_3_)_0.5_(OH)·0.11H_2_O (4)

Co^2+^ + F^−^ → CoF^+^ (5)

6H_2_O + (CH_2_)_6_N_4_ → 6HCHO + 4NH_3_ (6)

H_2_O + NH_3_ → OH^−^ + NH_4_^+^ (7)

CoF^+^ + OH^−^ → Co(OH)F (8)

6Co(OH)F + O_2_ → 2Co_3_O_4_ + 6HF (9)

6[Co(CO_3_)_0.5_(OH)·0.11H_2_O] + O_2_ → 3CO_2_ + 3.66H_2_O + 2Co_3_O_4_ (10)

**Figure 2:**
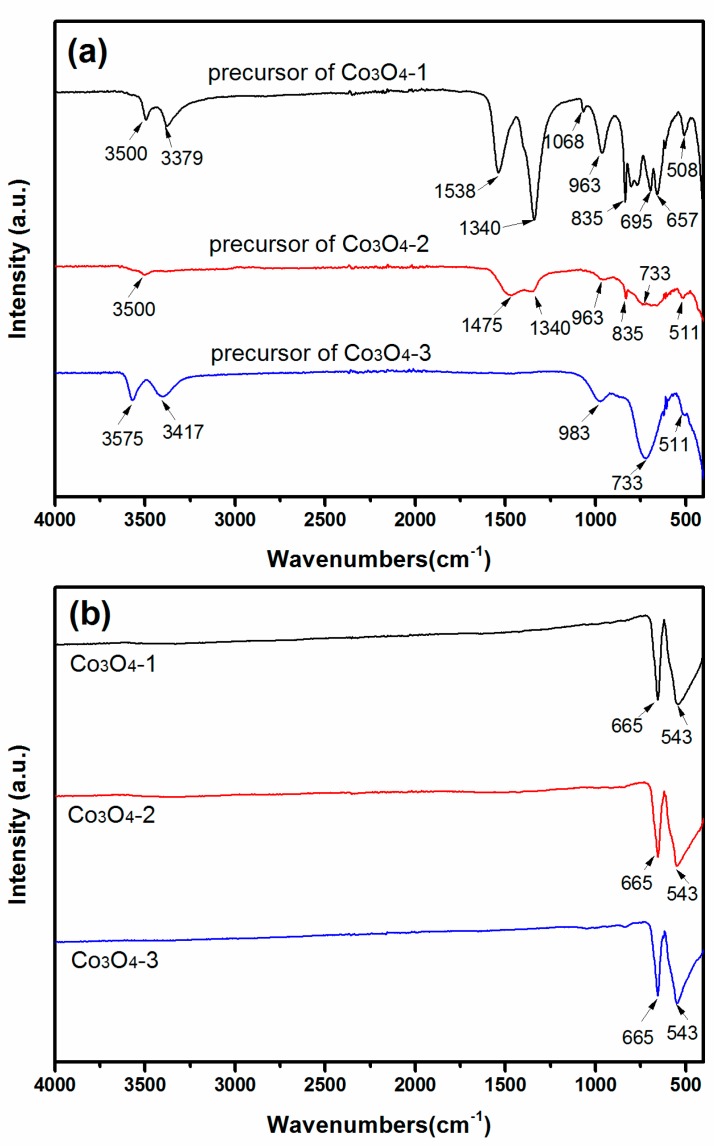
Typical FTIR spectra of the (**a**) precursors and (**b**) obtained Co_3_O_4_ products.

The FT-IR study also affirms the formation of precursors and the final Co_3_O_4_ samples. Figure 2 exhibits the FTIR spectra of Co_3_O_4_-1, Co_3_O_4_-2, and Co_3_O_4_-3, and their corresponding precursors. For the precursor of Co_3_O_4_-1, whose chemical composition is Co(CO_3_)_0.5_(OH)·0.11H_2_O, the peaks at 3500 and 3379 cm^−1^ are respectively ascribed to the O–H stretching mode of H_2_O and the bond between O–H groups and CO_3_^2−^. The bands observed at 1538 and 1340 cm^−1^ are assigned to stretching vibration *ν*(OCO_2_) and *ν*(CO_3_) respectively. However, compared with another two curves, to some extent, the strength of *ν*(OCO_2_) and *ν*(CO_3_) is weakened with the presence of Co(OH)F. The remaining weak peaks at 1068, 835, 733 and 695 cm^−1^ can respectively be *ν*(C=O), *δ*(CO_3_), *δ*(OCO), and *ρ*(OCO) [28,29,30,31]. The peaks that appeared at 963 and 511 cm^−1^ are ascribed to *δ*(Co–OH) and *ρ*_w_(Co–OH) bending modes, respectively [28,29,30,31]. For the precursor of Co_3_O_4_-3, whose chemical composition is Co(OH)F, the peak at 3575 cm^−1^ is assigned to the O–H stretching mode too. The shoulder vibration at 3417 cm^−1^ corresponds to the O–H groups interacting with fluoride anions. In addition, no any other band related to carbonate anions can be found (Figure 2a). After annealing treatment, the bands of precursors disappear and two very strong peaks are centered at 665 and 543 cm^−1^ characteristic of Co_3_O_4_ are noticed (Figure 2b). The former peak at 665 cm^−1^ corresponds to Co^2+^–O bond [30]. The other peak at 543 cm^−1^ can be ascribed to Co^3+^–O bond [31]. The results verified the formation of Co_3_O_4_ under thermal degradation, which coincides with the XRD patterns.

**Figure 3:**
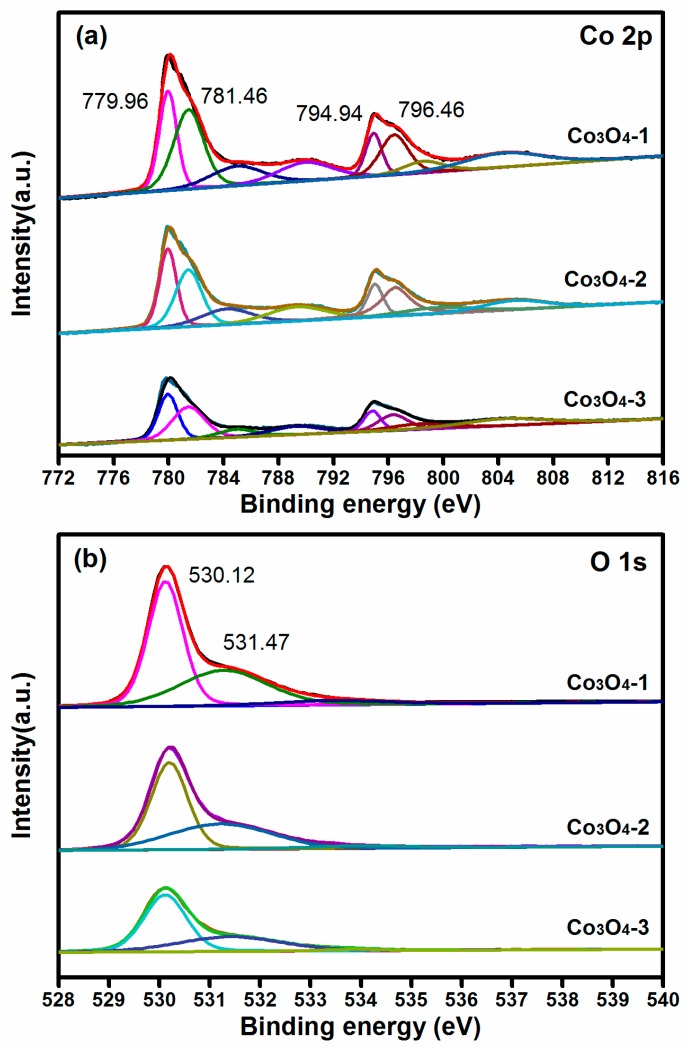
XPS analyses of (**a**) Co 2p and (**b**) O 1s for Co_3_O_4_-1, Co_3_O_4_-2 and Co_3_O_4_-3 nanocrystals.

To get more information about the chemical states of elements in prepared Co_3_O_4_ samples, XPS analysis was then performed. Figure 3a shows the Co 2p peaks of Co_3_O_4_-1, Co_3_O_4_-2, and Co_3_O_4_-3, respectively. The curve of Co 2p shows two spin-orbit doublets of Co 2p1/2 at 779.96 and 794.94 eV attributed to Co^3+^, and two spin-orbit doublets of Co 2p3/2 at 781.46 and 796.46 eV belonging to Co^2+^. The intensity of the peaks shows downward course from Co_3_O_4_-1 to Co_3_O_4_-3. Figure 3b depicts the O1s spectra of the prepared Co_3_O_4_ samples, the large O 1s peak at 530.12 eV is attributed to the lattice oxygen (LO) in Co_3_O_4_ crystals and the small O 1s peak at 531.47 eV represents the oxygen vacancies (OV) on the surface of Co_3_O_4_ [32,33,34]. The intensity of the oxygen vacancy also shows a decreased trend from Co_3_O_4_-1 to Co_3_O_4_-3 (Table 2). The more oxygen vacancies indicate a higher possibility for the exposure of active sites [35].

**Figure 4:**
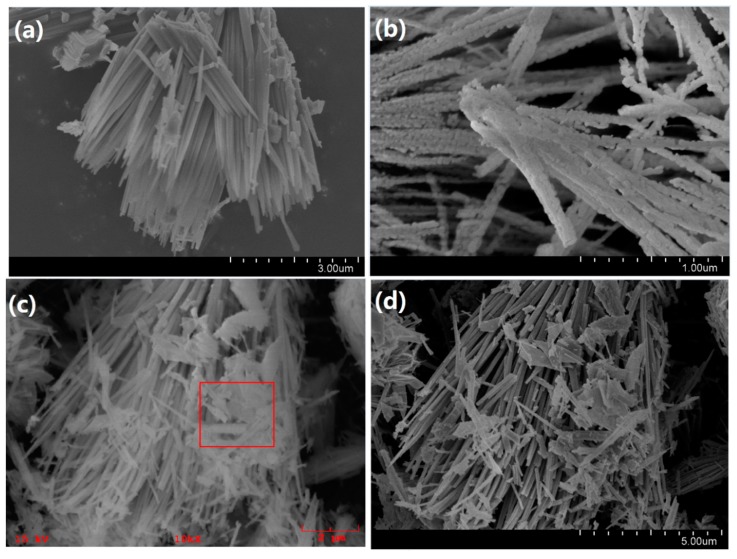

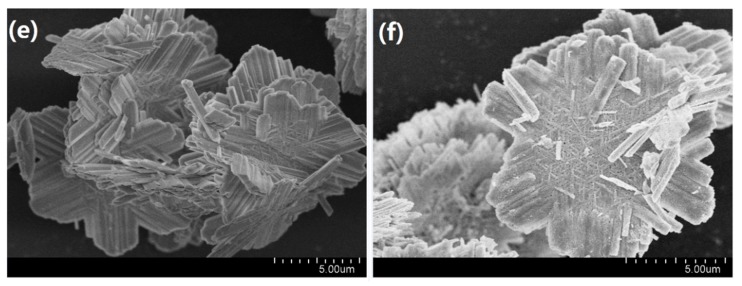
Typical FE-SEM images of (**a**) Co(CO_3_)_0.5_(OH)·0.11H_2_O precursor; (**b**) Co_3_O_4_-1; (**c**) the mixture of Co(CO_3_)_0.5_(OH)·0.11H_2_O and Co(OH)F; (**d**) Co_3_O_4_-2; (**e**) Co(OH)F precursor; and (**f**) Co_3_O_4_-3.

**Figure 5:**
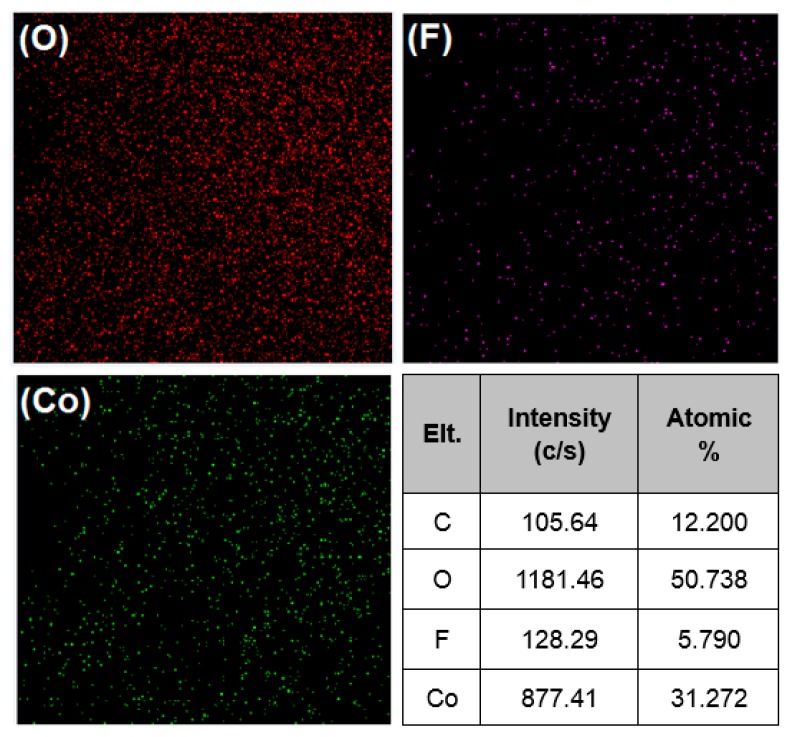
SEM-EDS elemental mapping (C, O, F, and Co) of the precursor of Co_3_O_4_-2.

The general morphology of the precursors and the obtained Co_3_O_4_ products was further explored using FE-SEM, as shown in Figure 4. The Co(CO_3_)_0.5_(OH)·0.11H_2_O precursor exhibited a nanorod structure, with a smooth surface (Figure 4a). The average length of the nanorods is in the range of 3–5 μm. The Co(OH)F precursor showed gear-like nanosheets, with an uneven surface (Figure 4e). The average thickness of Co(OH)F nanosheets is about 0.025 μm and the typical diameter of the nanosheets is in the range of 8‒12 μm. Figure 4b depicts the SEM image of the mixture of Co(CO_3_)_0.5_(OH)·0.11H_2_O nanorods and Co(OH)F nanosheets. When CO(NH_2_)_2_ was used as the hydrolysis reagent, both Co(CO_3_)_0.5_(OH)·0.11H_2_O and Co(OH)F precursors were generated in the same time with the existence of F. However, from Figure 4c, it is noteworthy that the quantity of nanorods is far more than nanosheets, which indicates that the growth rate of Co(CO_3_)_0.5_(OH)·0.11H_2_O is faster than the growth rate of Co(OH)F. The elemental mapping analysis in the selected area of Figure 4c further confirmed the generation of Co(OH)F. Figure 5 suggests that the F element was evenly dispersed within the Co(OH)F sample, with a calculated atomic amount of ca. 5.8%. After annealing the precursors, the morphology of obtained Co_3_O_4_-1, Co_3_O_4_-2, and Co_3_O_4_-3 samples were also characterized using FE-SEM, as shown in Figure 4b,d,f. It was worthwhile mentioning that the products still maintained the similar morphology with their precursors after the annealing treatment. Contrary to the precursors, the Co_3_O_4_ displayed rough and porous surfaces, which might be due to the abscission of attached OH ions during the calcination process. The difference of morphology may lead to the difference of specific surface area of Co_3_O_4_. Based on the BET data, it could be found that the rod-like Co_3_O_4_ possessed larger specific surface area than sheet-like Co_3_O_4_. The relatively large specific surface area of rod-like structure may be beneficial to the exposure of active sites and in the meantime facilitate the contact of Co_3_O_4_ to the targeted chemicals.

### 2.2. Hydrazine Chemical Sensor Studies of Co_3_O_4_ Modified Electrodes

**Figure 6:**
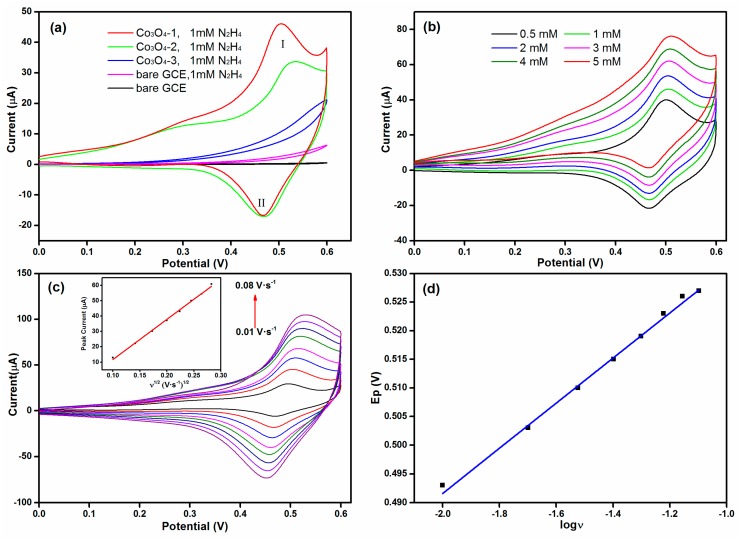
(**a**) Cyclic voltammograms for different modified electrodes in the presence of 1 mM hydrazine at a scan rate of 0.02 V·s^−1^; (**b**) Cyclic voltammograms of Co_3_O_4_-1/GCE with different hydrazine concentrations at a scan rate of 0.02 V·s^−1^; (**c**) Cyclic voltammograms of Co_3_O_4_-1/GCE with different scan rates (From 0.01 to 0.08), inset shows the anodic peak current vs. the square root of scan rate (*ν*^1/2^); and (**d**) the anodic peak potential (Ep) vs. log *ν*.

To prepare the hydrazine sensor, Co_3_O_4_ nanomaterials were coated on the surface of GCE. The electrocatalytic activity of Co_3_O_4_ nanomaterials towards hydrazine was firstly investigated by cyclic voltammetry technique. Figure 6a shows the cyclic voltammogram (CV) of bare GCE and Co_3_O_4_/GCE in the presence of 1 mM hydrazine in 0.1 M NaOH electrolyte at a scan rate of 0.02 V·s^−1^. It is apparent that no matter whether hydrazine exists, the bare GCE does not exhibit any redox peak in 0 to 0.6 V, just the current elevated when hydrazine added. This result indicates the low catalytic activity of bare GCE. However, for these three kinds of Co_3_O_4_ modified electrodes, there are significant differences among them. For Co_3_O_4_-3 (obtained from Co(OH)F) modified electrode, no peak could be observed when it was tested. However, for Co_3_O_4_-2 (obtained from the mixture) and Co_3_O_4_-1 (obtained from Co(CO_3_)_0.5_(OH)·0.11H_2_O) modified electrodes, an oxidation peak (I) apparently emerged at around 0.50 V. The observed CV response also exhibited reversible nature as it showed a reduction peak (II) at 0.46 V during the reverse sweep. Among them, Co_3_O_4_-1 possesses the best electrochemical activity towards hydrazine oxidation while Co_3_O_4_-3 has the poorest electrochemical activity towards hydrazine. This result suggests that F has a negative effect on the performance of Co_3_O_4_. We believe that the performance of Co_3_O_4_ should be related to their crystallinity and specific surface area. According to the XPS analysis, with increasing the amount of F in the synthesis process, the oxygen vacancy of their final product (Co_3_O_4_) shows a decreased trend. The addition of F allows the precursor to grow more regularly but cause the decrease of the specific surface area, resulting in relatively less active sites and worse performance of Co_3_O_4_ [36]. The possible reactions on Co_3_O_4_ electrode can be expressed as the following Equations (11)–(13).

OH^−^ + H_2_O + Co_3_O_4_ → 3CoOOH + e^−^ (11)

OH^−^ + CoOOH → H_2_O + CoO_2_ + e^−^ (12)

4CoO_2_ + N_2_H_4_ → 4CoOOH + N_2_ (13)

The influence of hydrazine concentration and the scan rates on the performance of modified electrode was then investigated using the Co_3_O_4_-1 sample. Figure 6b exhibits the cyclic voltammograms of Co_3_O_4_-1 modified GCE with different hydrazine concentrations at a scan rate of 0.02 V·s^−1^. With increasing the hydrazine concentration from 0.5 to 5 mM, the current displays a growth trend. The simultaneous response reveals that the fabricated Co_3_O_4_-1-based sensor can be used for the effective determination of hydrazine. Figure 6c depicts the cyclic voltammograms of Co_3_O_4_-1 modified GCE with 1 mM hydrazine at different scan rates ranging from 0.01 to 0.08 V·s^−1^. The inset of Figure 6c shows that the peak current (Ip) also increases synchronously with the scan rate. The relationship between Ip and the scan rate was further calculated based on the Randles–Sevcik equation [37]. The equation can be expressed as Ip = 261.3 *ν*^1/2^ − 14.4 (R^2^ = 0.997). The negative intercept may be due to the adsorption of the N_2_H_4_ occurred on the electrode surface, which indicates that the electrode reaction is not a single diffusion-controlled process [38]. Moreover, it is notable that the peak potential shift towards positive potential with increasing the scan rate. Figure 6d exhibits the linear relation between the peak potential (Ep) and log (*ν*), implying the irreversible oxidation of hydrazine at the surface of Co_3_O_4_-1/GCE.

**Figure 7:**
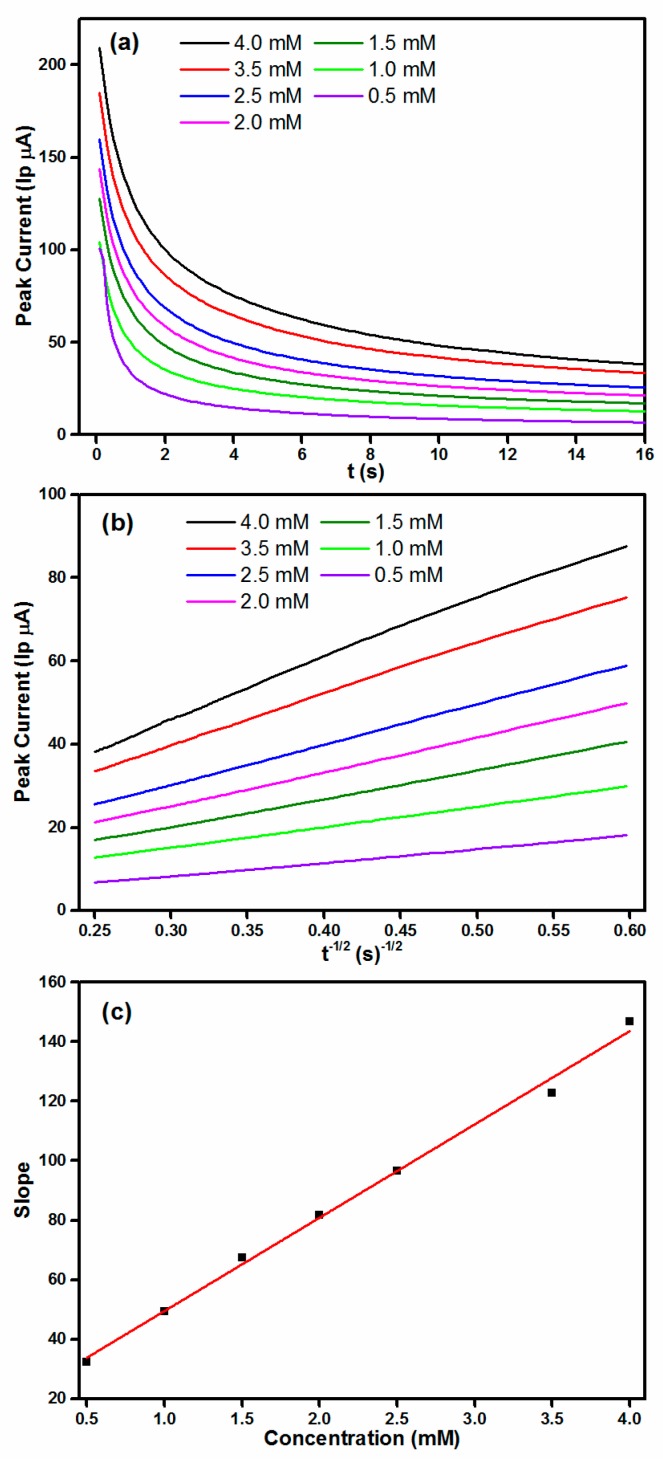
(**a**) Chronoamperograms obtained at Co_3_O_4_-1/GCE with different concentrations of hydrazine in 0.1 M NaOH. Applied potential was 0.50 V; (**b**) Plot of current versus *t*^−1/2^; and (**c**) The plot of slopes obtained from straight lines versus concentration of hydrazine.

Figure 7a displays the chronoamperometric response of Co_3_O_4_-1/GCE with different concentration of hydrazine. The transient currents decayed with prolonging the time, also revealing the diffusion-controlled process of hydrazine electrooxidation. The peak current exhibited linear relationship with *t*^−1/2^ (Figure 7b). In addition, the slope of the line increased with increasing the hydrazine concentration (Figure 7c). Thus, the diffusion coefficient of hydrazine (*D*) could be calculated via Cottrell’s equation:

Ip = *nFAD*^1/2^*Cπ*^−1/2^*t*^−1/2^ (14)

In Equation (14), *n*, *F* (C∙mol^−1^), *A* (cm^2^), *C* (mol∙cm^−3^) respectively represents the number of involved electron transfer, the Faraday constant (96,485), the surface area of GCE, and the dosage of hydrazine. The slopes of the obtained linear lines were plotted against the hydrazine concentrations (Figure 7c). Based on this plot, *D* was determined to be 1.66 × 10^−5^ cm^2^∙s^−1^, which is consistent with the previous report [39].

### 2.3. Amperometric Detection of Hydrazine Using Co_3_O_4_ Modified Electrodes

**Figure 8:**
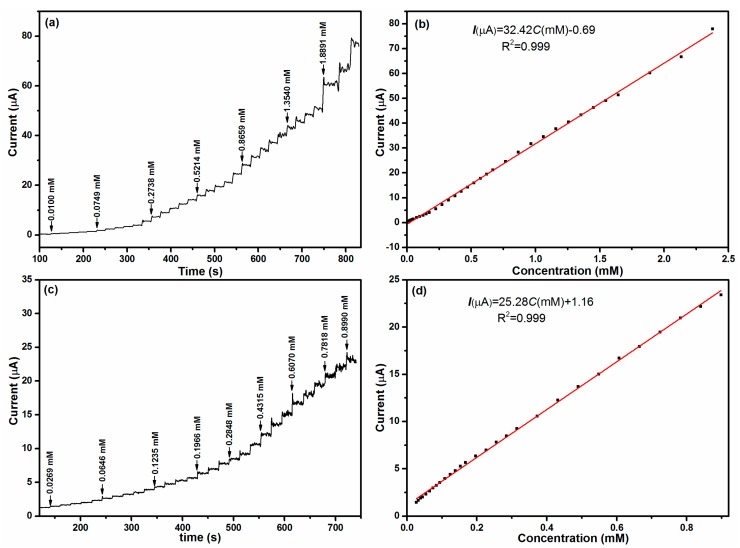
Amperometric responses of (**a**) Co_3_O_4_-1 and (**c**) Co_3_O_4_-2 modified electrodes with successive addition of hydrazine into 0.1 M NaOH; (**b**,**d**) the linear relationships between current vs. hydrazine concentration of Co_3_O_4_-1 and Co_3_O_4_-2 modified electrodes.

The Co_3_O_4_-1 and Co_3_O_4_-2 modified electrodes were then used as a sensor for detection of hydrazine. The work potential was set at 0.50 V. For comparison, the amperometric responses of Co_3_O_4_-1 and Co_3_O_4_-2 electrodes are displayed. From Figure 8a,c, it is apparent that with the successive addition of hydrazine to a stirred solution, the anodic current increases gradually. When an aliquot of hydrazine was dropped into the stirred NaOH solution, the amperometric responses of the Co_3_O_4_-1 modified electrode achieved a steady state within 2 s, which is faster than the Co_3_O_4_-2 modified electrode. On the other hand, the magnitudes of the response current of the Co_3_O_4_-1 modified electrode is also larger than the Co_3_O_4_-2 modified electrode at the same condition. These results suggest that the Co_3_O_4_-1 modified electrode has better electrochemical performance than the Co_3_O_4_-2 modified electrode. When the hydrazine concentration exceeds a certain range, the response currents will no longer increase, but turn to be a declining trend. This phenomenon indicates that the hydrazine concentration exceeds the critical value of linear range. In order to further distinguish the difference between the two electrodes, mathematic fitting was utilized to calculate the sensitivity, linear response range and the detection limit. Figure 8b,d exhibit the liner relationship between hydrazine concentration and response current. For the Co_3_O_4_-1 modified electrode, the equation can be explained as *I*(μA) = 32.42 *C*(mM) − 0.69 (R^2^ = 0.999), while for the Co_3_O_4_-2 modified electrode, the equation can be presented as *I*(μA) = 25.28 *C*(mM) + 1.160 (R^2^ = 0.999). The plots also displayed a linear relationship with the hydrazine concentration in the range of 0.010 to 2.380 mM and 0.027 to 0.890 mM, respectively. For Co_3_O_4_-1 and Co_3_O_4_-2, the sensitivity that was found to be 32.42 μA·mM^−1^ and 25.28 μA·mM^−1^, respectively. The detection limit for the Co_3_O_4_-1 and Co_3_O_4_-2 fabricated hydrazine sensors were calculated to be 9.73 μM and 10.74 μM (S/N = 3).

Table 3 summarizes the electrochemical parameters of some reported N_2_H_4_ sensors. Compared with them, the Co_3_O_4_-1/GCE and Co_3_O_4_-2/GCE exhibit rather high sensitivity and wide linear range. The performance of Co_3_O_4_-1/GCE is better than Co_3_O_4_-2/GCE. These results reveal that the existence of F in the synthesis of Co_3_O_4_ has a great impact on its electrochemical performance. To explore the influence of surface area of the obtained Co_3_O_4_ samples on their electrochemical performances, the surface area normalized current and sensitivity of Co_3_O_4_-1(obtained bare Co(CO_3_)_0.5_(OH)·0.11H_2_O) and Co_3_O_4_-2 (obtained the mixture of Co(CO_3_)_0.5_(OH)·0.11H_2_O and Co(OH)F) were shown as Table 4. The Co_3_O_4_-1/GCE and Co_3_O_4_-2/GCE exhibit no significant difference in surface area normalized current and surface area normalized sensitivity, which implies that the specific surface area is one of crucial factors for the electrochemical performances of Co_3_O_4_. As above-mentioned, the amount of NH_4_F is inversely related to the specific surface area. The dosage of NH_4_F during the hydrothermal process affects the specific surface area of the products directly and therefore causes the difference in their electrochemical performances. To obtain the highly active Co_3_O_4_-based modified electrodes for hydrazine detection, F should be avoided, although in many cases the F species cannot be detected by the XRD and FTIR analysis. However, the mechanism on how the remaining F affects the electrochemical activity of Co_3_O_4_ is still under investigation.

### 2.4. Selectivity, Reproducibility and Stability Tests

**Figure 9:**
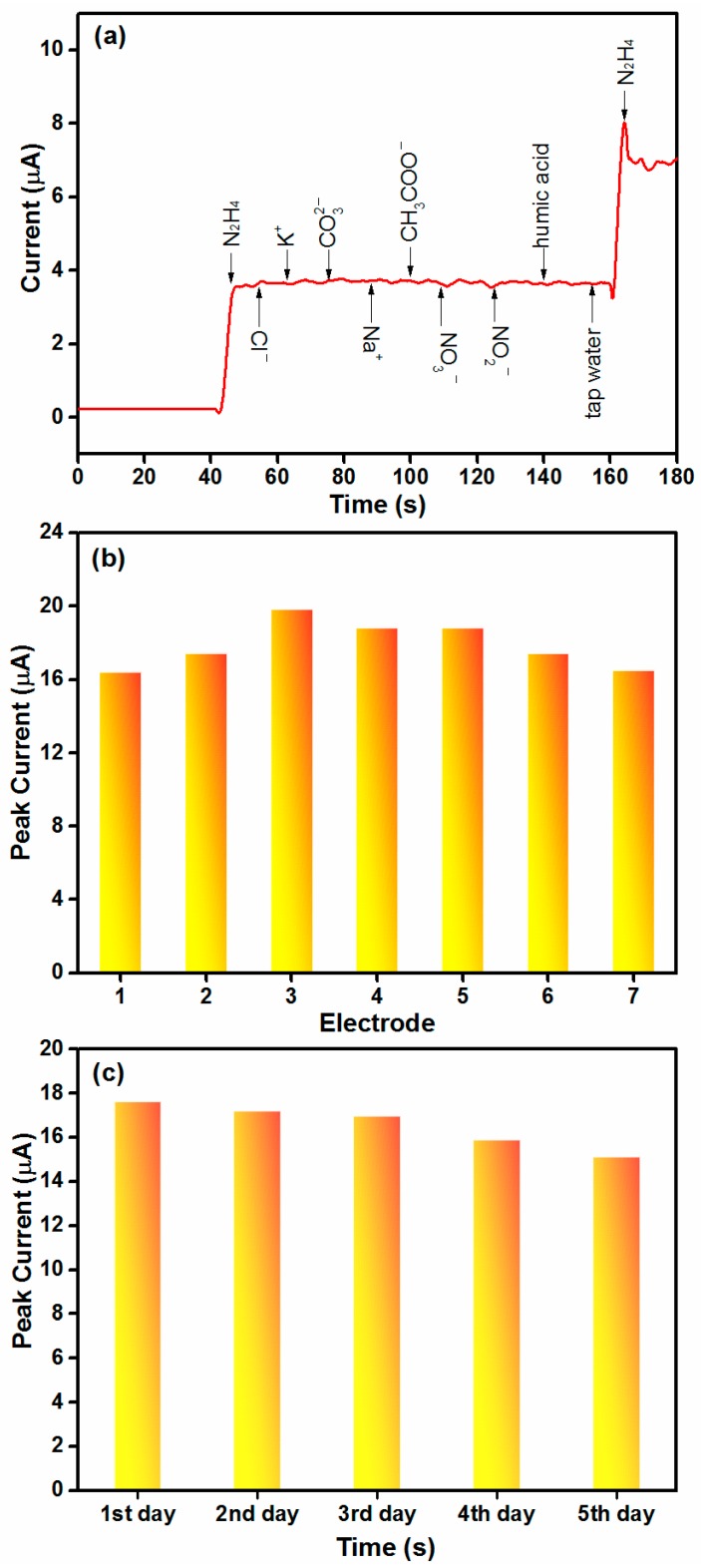
(**a**) The amperometric response to the addition of hydrazine with interfering species including Cl^−^, CO_3_^2−^, NO_3_^−^, NO_2_^−^, CH_3_COO^−^, K^+^, Na^+^, tap water, and humic acid; (**b**) Peak currents of seven electrodes evaluated in 1 mM hydrazine; and (**c**) The peak current of Co_3_O_4_-1/GCE after being stored at room temperature for five days.

Selectivity and stability are two of key parameters to evaluate performance of chemical sensors. Thus, the selectivity and stability of the Co_3_O_4_-1-based hydrazine sensor were also explored. Figure 9a exhibits the i–t curve response of hydrazine and interferent (Cl^−^, CO_3_^2−^, NO_3_^−^, NO_2_^−^, CH_3_COO^−^, K^+^, Na^+^, tap water, and humic acid). When 0.1 M N_2_H_4_ (10 μL) was injected to the NaOH solution, a quick response can be detected. However, when the same dosage of interfering species is added to the electrolyte, no obvious current response could be observed, suggesting the good selectivity of Co_3_O_4_-1/GCE for N_2_H_4_ detection. To evaluate the reproducibility, seven different glassy carbon electrodes were prepared via the same modification step. The relative standard deviation value of peak current towards 1 mM hydrazine was found to be 7.23% (Figure 9b). To test the stability, the electrode was stored for five days in ambient conditions. Figure 9c displays the peak current of Co_3_O_4_-1/GCE within five days. The value of peak current shows a declining trend with prolonging the time, but the peak current can still reach 86% of its initial response after being stored for five days. The obtained result suggests the long time stability of Co_3_O_4_-1/GCE.

### 2.5. Real Sample Test

In order to evaluate the validity of the proposed method, the Co_3_O_4_-1/GCE was applied for the detection of hydrazine in different water samples which prepared by adding known amounts of hydrazine in water samples, the results are listed in Table 5. When a known amount of hydrazine was added to distilled water, tap water, and river water, quantitative recoveries of 99.77–102.79%, 98.33–101.63%, 98.63–99.30% were obtained respectively. All the results revealed the feasibility of the proposed electrode in the determination of hydrazine in water samples.

## 3. Materials and Methods

### 3.1. Synthesis of Co_3_O_4_ Nanostructures

All the precursors were synthesized using a straightforward hydrothermal process. The detailed synthesis procedures were described as follows. 1.455 g (5 mmol) of Co(NO_3_)_2_·6H_2_O and 0.601 g (10 mmol) of CO(NH_2_)_2_ were mixed in 50 mL DI water and stirred continuously for 10 min. The obtained mixture solution was moved into a 100 mL autoclave and then hydrothermally at 95 °C for 24 h. The attained precipitates were concentrated via centrifugation, and repeatedly rinsed with absolute ethanol and distilled water and subsequently dried at 60 °C. The dried powder was the precursor for Co_3_O_4_-1, being Co(CO_3_)_0.5_(OH)·0.11H_2_O. The precursor for Co_3_O_4_-2 was prepared using the similar way except extra addition 0.370 g (10 mmol) of NH_4_F, being Co(CO_3_)_0.5_(OH)·0.11H_2_O and Co(OH)F mixture. The precursor for Co_3_O_4_-3 was synthesized similarly to the precursor of Co_3_O_4_-2 but changing the urea to hexamethylenetetramine (C_6_H_12_N_4_, HMT), being pure Co(OH)F. The detailed synthesized conditions are summarized in Table 1. To obtain the Co_3_O_4_ products, all the precursors were annealed at 400 °C for 4 h. For ease of description, the products obtained from bare Co(CO_3_)_0.5_(OH)·0.11H_2_O, pure Co(OH)F, and their mixture were designated as Co_3_O_4_-1, Co_3_O_4_-3, and Co_3_O_4_-2, respectively.

### 3.2. Electrode Modification

Before modification, the prepared glassy carbon electrode (GCE) was respectively polished with 1.0, 0.3, 0.05 μm alumina powder for 10 min, and then rinsed with distilled water followed by drying under ambient conditions. The obtained homogeneous slurries containing 5 mg Co_3_O_4_, 50 μL of Nafion solution (5 wt. %, DuPont 520, Wilmington, DE, USA), and 1 mL of ethanol was the mixture of all chemical together via sonication for 30 min. The Co_3_O_4_ modified GCE was produced by transferring 5 μL of the above attained homogeneous slurry on the GCE, and followed dried at ambient temperature.

### 3.3. Characterization of Samples

XRD spectra were acquired from Shimadzu XRD‒7000 (Shimadzu, Kyoto, Japan) diffractometer (2θ = 10°‒70°). Molecular speciation of the samples was examined by Bruker VERTEX 70 FT-IR spectrophotometer (Bruker, Billerica, MA, USA) in the range of 4000–400 cm^−1^. XPS analysis was performed on a Thermo Scientific Escalab 250Xi instrument (Thermo Scientific, Waltham, MA, USA). The specific surface area (SSA) analysis was conducted by using Builder SSA-7000 (Beijing Builder Electronic Technology Co., Ltd, Beijing, China). SEM analysis was performed on a Hitachi SU8010 (Hitachi, Tokyo, Japan) field emission scanning microscope.

## 4. Conclusions

In summary, three types of Co_3_O_4_ samples were prepared and utilized as electrode materials for hydrazine detection. XRD analyses demonstrated that the precursors for Co_3_O_4_-1, Co_3_O_4_-2, and Co_3_O_4_-3 were Co(CO_3_)_0.5_(OH)·0.11H_2_O, the mixture of Co(OH)F and (Co(CO_3_)_0.5_(OH)·0.11H_2_O, and Co(OH)F, respectively. SEM analyses showed that these three Co_3_O_4_ samples possess different morphologies. The existence of F in their precursors was confirmed using SEM-EDS elemental mapping. Cyclic voltammetry results revealed that the electrochemical activity of Co_3_O_4_ decreased with the increase of F content in precursors. Furthermore, the prepared Co_3_O_4_-1 and Co_3_O_4_-2 were used to fabricate hydrazine chemical sensor. The results indicated that the Co_3_O_4_-1-based hydrazine sensor possessed a high sensitivity of 32.42 μA·mM^−1^, a low detection limit of 9.7 μΜ (S/N = 3), and a wide linear range from 0.010 to 2.380 mM. All these observed parameters were much better than those of the Co_3_O_4_-2 or Co_3_O_4_-3-based hydrazine sensors. The obtained results show that the fabricated hydrazine sensor also has good selectivity, great reproducibility and longtime stability.

## Figures and Tables

**Table 1:** Synthesis of Co_3_O_4_ nanocrystals with different addition of NH_4_F.

Raw Materials (mmol)			Precursor	Calcination (400 °C)	Crystallinity (%)	SSA (m^2^∙g^−1^)
Co(NO_3_)_2_	NH_4_F	CO(NH_2_)_2_				
5	0	10	P1 ^1^	Co_3_O_4_-1	73.87	25.83
5	5	10	P1 and P2	Co_3_O_4_	80.34	21.20
5	10	10	P1 and P2	Co_3_O_4_-2	84.24	18.44
5	15	10	P1 and P2	Co_3_O_4_	88.05	14.55
5	20	10	P1 and P2	Co_3_O_4_	90.46	12.44
5	20	HMT	P2 ^1^	Co_3_O_4_-3	91.62	10.57

^1^ P1 and P2 represents Co(CO_3_)_0.5_(OH)·0.11H_2_O and Co(OH)F, respectively.

**Table 2:** XPS data of Co and O species of Co_3_O_4_ derived from different precursors.

Sample	Amount of Oxygen Species			
	Oxygen Vacancy (OV)		Lattice Oxygen (LO)	
	at. %	BE (eV)	at. %	BE (eV)
Co_3_O_4_-1	44.9	530.12	55.1	531.47
Co_3_O_4_-2	43.8	530.12	56.2	531.47
Co_3_O_4_-3	41.8	530.12	58.2	531.47

**Table 3:** Comparison of electrochemical parameters of various nanomaterials-based hydrazine sensor.

Electrode Materials	Sensitivity (μA∙μM^−1^)	Detection Limit (μM)	Linear Range (mM)	Ref.
Ru-complex films	–	8.5	0.010–10	[40]
Nano-Au/Ti	1.117	42	0.5–4	[41]
Nickel tetrasulfonated phthalocyanine	0.0079	10	0.1–0.6	[42]
MWCNTs/Chlorogenic	4.1 μA∙mM^−1^∙cm^−2^	8	0.0025–0.5	[43]
GO/CTS/Pt	104.6 μA∙mM^−1^∙cm^−2^	3.6	0.02–1	[44]
Co_3_O_4_-1	32.42	9.73	0.010–2.38	This work
Co_3_O_4_-2	25.28	10.74	0.027–0.890	

**Table 4:** The surface area normalized current and sensitivity of the obtained Co_3_O_4_ samples.

Materials	Current (μA)	Sensitivity (μA∙mM^−1^)	SSA (m^2^∙g^−1^)	Surface Area Normalized Current (μA∙m^−^^2^∙g)	Surface Area Normalized Sensitivity (μA∙mM^−1^∙m^−^^2^∙g)
Co_3_O_4_-1	46.05	32.42	25.83	1.78	1.26
Co_3_O_4_-2	33.58	25.28	18.44	1.81	1.37
Co_3_O_4_-3	-	-	10.57	-	-

**Table 5:** The real sample analysis of Co_3_O_4_-1-based hydrazine sensor using recovery method.

Sample	Hydrazine Added (μM)	Hydrazine Founded (μM)	Recovery
**Distilled Water**	10	10.28	102.79%
	20	19.98	99.88%
	50	49.88	99.77%
**Tap Water**	10	10.16	101.63%
	20	19.63	98.14%
	50	49.16	98.33%
**River Water**	10	9.86	98.63%
	20	19.51	97.56%
	50	49.65	99.30%
